# Providers’ Practices and Associated Factors in Educating Pregnant Women on the Prevention of Maternal Anemia During Antenatal Care Visits in Ujiji Municipality, Kigoma Region, Tanzania

**DOI:** 10.3390/healthcare13030327

**Published:** 2025-02-05

**Authors:** Fredy Marwa, Eveline Konje, Theckla Tupa, Mlemile Gwimile, Namanya Basinda

**Affiliations:** 1 Tanzania Health Promotion Support, Dar es Salaam P.O. Box 32605, Tanzania; fmarwa@thps.or.tz; 2 School of Public Health, Catholic University of Health and Allied Sciences, Mwanza P.O. Box 1464, Tanzania; ekonje28@bugando.ac.tz; 3 Weill-Bugando School of Medicine, Catholic University of Health and Allied Sciences, Mwanza P.O. Box 1464, Tanzania; thecklatupa1@gmail.com (T.T.); mlemile75@gmail.com (M.G.)

**Keywords:** antenatal care, providers’ practices, maternal anemia, educating pregnant women, client exit interview, Ujiji Municipality

## Abstract

Background: Anemia in pregnancy is among the preventable severe public health problems, responsible for around 14.5% of maternal mortality in Tanzania. Antenatal visits are among the pillars that aid in reducing the burden of anemia in pregnancy. However, there are discrepancies in adherence and performance across different domains of Antenatal care (ANC) by healthcare workers. Therefore, this study examined providers’ practices and associated factors in educating pregnant women on preventing maternal anemia during antenatal care visits in Kigoma and Ujiji Municipal Council in the Kigoma region. Methods: This was a cross-sectional design involving a total of 430 client exit interviews across 18 facilities. The association between the provision of maternal anemia education and other factors such as health facility level, age group, sex, economic activities, and ANC visitation was analyzed using multivariate logistic regression. *p*-values less than 0.05 were considered significant throughout the study. Results: The study revealed that most participants (70.4%) reported receiving anemia information during ANC visits. The most common topics were insecticide-treated nets (ITNs) (76%) and types of food to prevent anemia (74%), while 20.8% and 24.8% reported receiving information about worm infestation and deworming, respectively. Only 34.4% (95%CI: 26.5–43.3%) of pregnant women were informed of at least five topics. Conclusions: The study reveals limited education provision on anemia among pregnant women. There is a discrepancy in topic coverage among facilities and individuals.

## 1. Introduction

Anemia in pregnancy is a disease of major concern globally, classified as a severe public health problem, with its prevalence adding up to 40% of pregnant women worldwide [1,2]. Anemia is implicated in several severe outcomes, including high rates of maternal deaths, perinatal deaths, preterm delivery, and cesarean deliveries for pregnant women with anemia [3,4,5]. Anemia in pregnancy is linked to low birth weight and anemia in children born to mothers with anemia [4,6,7]. Anemia is quantified as hemoglobin (Hb) levels below 11.0 g/dL among pregnant women and subcategorized as severe anemia when Hb is below 7.0 g/dL [8,9]. The situation is more adverse in low- and middle-income countries, where the prevalence of anemia in pregnancy is 56%. The prevalence is highest in sub-Saharan countries, accounting for 57% of pregnant women having anemia. In Tanzania, anemia rates range from 16% to 64%; as per multiple studies, this still warrants it as an issue of public health concern [10,11,12]. In Tanzania, anemia is a significant, critical indirect cause of 14.5% of maternal mortality [13].

Studies have identified various causes of anemia among pregnant women, such as poor iron supplementation, malnutrition, endemic diseases like malaria and worm infestations, early marriage, and ill spacing between two pregnancies [14]. Initiation of interventions such as deworming, iron supplementation, and insecticide-treated mosquito nets (ITNs) has brought a relatively small decline in anemia in females of reproductive age, from 48% in 2005 to 45% in 2015 [15]. These interventions are delivered during antenatal visits [15,16,17].

Antenatal visits are among the pillars that aid in reducing the burden of anemia in pregnancy. Poor attendance at antenatal visits among pregnant women is a major factor contributing to the ongoing burden of anemia in pregnancy [18]. A recent study in Tanzania shows that about 98% of women between the ages of 15 and 49 have received Antenatal Care (ANC) services from a skilled provider [17]. However, studies indicate discrepancies in adherence and performance across different domains of ANC by healthcare workers, with a high level of adherence observed in client history taking and HIV testing and counseling. In contrast, other domains are given less attention [19,20]. It is therefore essential to determine adherence and performance by healthcare providers to the teaching of the five components of ANC in Tanzania, with a focus on the local context [20]. Therefore, this study assessed how healthcare workers provide health education to pregnant women in terms of topic coverage regarding the prevention of anemia and the factors influencing this process.

## 2. Materials and Methods

This was a health facility-based cross-sectional study involving 430 client exit interviews (with twenty-three participants per facility across 18 facilities). The study population included pregnant women attending the antenatal care services in all the eighteen health facilities involved in this study.

The study was carried out in the Kigoma–Ujiji Municipal Council in the Kigoma region in Tanzania. Kigoma has six districts and eight councils. It is a region bordering Congo and Burundi, and according to the 2022 Tanzania National Census, Kigoma–Ujiji Municipal Council has a population of 243,216, based on estimates from 2015 [21,22]. Fishing and agriculture are the main economic activities [21]. The prevalence of anemia in pregnancy in Kigoma is 77.1%, which is the third highest [14]. Kigoma–Ujiji Municipal Council has 20 health facilities, all providing ANC services. On average, 484 pregnant women attended ANC at each facility in 2021. In addition, out of 343 existing healthcare providers, 11% work in reproductive and child health (RCH) units.

All facilities providing ANC services in Ujiji were involved in the study. At each facility, a list of women attending ANC services on the day of data collection was generated to create a sampling frame. A total of ten women were selected from the day’s attendance. First, the total number of women on the day was divided by 10 to determine the K value. Then, the first woman was selected by lottery, then every K-th woman was selected until 10 women were included in the day’s data collection at each facility.

A questionnaire was used to collect data from exit interviews. This questionnaire was developed based on the components of ANC care for anemia prevention guidelines [23]. The questionnaire assessed five elements of ANC care on anemia prevention, which included adherence to iron–folic acid supplementation, education on micronutrient fortification of commonly consumed local foods, proper use of insecticide-treated bed nets, adherence to IPT, adherence to deworming services, and optimal birth spacing of at least two years. It was then reviewed by three experts, who looked at the contents of the questions and made recommendations. The checklist was developed in English and then translated into Swahili. It was back-translated by another person conversant in both languages to ensure no loss of translation. The questionnaire was pretested in Nyamagana district to sort out wording issues or questions that were difficult to answer; thus, the questionnaire was examined to see if it could measure what it was meant to measure [24]. Data collection was done after the completion of the informed consent process.

Collected data was validated using the EpiInfo program and later exported to STATA version 15 (College Station, TX, USA) for analysis. Descriptive analysis was performed using frequency and percentage. The simple association between the provision of maternal anemia education and other factors, such as health facility level, age group, sex, economic activities, and ANC visitation, was explored, followed by multivariate logistic regression. Variables that were found to be significant at the bivariate level, using 0.05 as a level of significance, were included at the multivariate level. The odds ratio with a 95% confidence interval was calculated for both bivariate and multivariate associations. *p*-values less than 0.05 were considered significant throughout the study.

## 3. Results

### 3.1. Sociodemographic Characteristics of Respondents

The survey involved 430 women attending antenatal care clinics in preselected public health facilities in Ujiji Municipality. The majority of participants were aged 20–29 years, with 90% falling within the 20–39 age range. About 31% of the women had attained secondary or tertiary education. Most participants (44%) were self-employed and engaged in small businesses, while only 5% were employed by government institutions. Additionally, 28% of the women were visiting the clinic for the first time, and 11% had attended antenatal care clinics more than four times. For additional details, please refer to Table 1.

### 3.2. Provision of Anemia Health Education Components (Topics) During ANC Visits

The survey observed nine components of anemia education for pregnant women while attending ANC, including a general talk about anemia prevention, the minimum required hemoglobin level in pregnancy, consequences of anemia in pregnancy, types of food to prevent anemia, information about worm infestation, information about deworming, information about insecticide-treated nets (ITNs), information about intermittent prophylaxis treatment (IPT), and information about iron supplements. The type of food included examples of animal foods rich in iron, such as organ meat (liver, kidney, heart), flesh meat (beef, pork, lamb, rabbit, dog, chicken, duck), insects (insect larvae, senene, grasshoppers, ants (kumbikumbi)), fish and seafood (fresh fish, dried fish, canned fish, prawns, shrimps, seafood), and plants, including nuts (groundnuts, njugu) and legumes (beans, green beans, cowpeas, soya beans, and others). More importantly, the training should include foods that help the body absorb and use iron; for example, vitamin C-rich foods, such as fresh citrus fruits (guava, orange, lemons, apple, pawpaw, cabbage, cornflower, tomatoes, green vegetables, green papers, etc.). It should also include advice on avoiding beverages that decrease iron absorption when taken with meals, such as coffee.

The majority of participants reported receiving information about malaria prevention measures (ITNs and IPT) (77%), types of food to prevent anemia (75%), and general talk about anemia prevention (70%), while only 28% and 44% of pregnant women received information about worm infestation and deworming, respectively. Ideally, during the ANC visit, the service provider should provide education on anemia covering all topics. However, based on the exit survey, about 58.6% (95%CI: 53.9–63.2%) of pregnant women reported being informed of at least five topics. See Table 2.

### 3.3. Factors Associated with the Reported Provision of Anemia Health Education from the Exit Survey

The bivariate analysis was performed to determine factors associated with the reported provision of anemia health education during the current ANC clinic visit. In the bivariate analysis, secondary or above education level, occupation, and number of antenatal care visits were statistically associated with reporting being informed about anemia health education, whereas the age group of a participant was not associated. In the multivariate analysis, those attending ANC clinics more than twice showed higher odds of reporting the provision of anemia health education compared to those who attended for the first time. Similarly, the clients with secondary and tertiary education levels showed two times higher odds of reporting the provision of anemia health education during antenatal visits than those with no formal and primary education level. See Table 3.

## 4. Discussion

This study explored the health education coverage on anemia provided to women attending ANC in health facilities. The study benchmarked the components of anemia prevention education provided to women against the topics that should be provided according to the ANC guideline [23]. Therefore, the findings of this study show the gap in education coverage compared to what is supposed to be provided.

The study revealed that 58.6% of participants received education on anemia. This is notably low and does not serve the purpose, given the fact that ANC is an area of intervention for the prevention of anemia in pregnancy. This implies that despite the high percentages of antenatal care visits, these visits do not reflect the quality of antenatal care given [25]. The provision of education has been shown to improve hemoglobin levels among pregnant women [26]. Hence, this situation presents one of the most significant missed opportunities to correct anemia and reduce maternal mortality. The quality of ANC and its improvement could significantly reduce the burden of anemia.

Coverage of educational topics on anemia varied among respondents. The provision of education covered different topics with varying coverage, ranging from 20.8% to 76.8%. The difference in coverage of topics has been highlighted in other studies due to different enablers and bottlenecks [27]. A study carried out in Uganda, Tanzania, and Burkina Faso uncovered irregularities in group education sessions, offered on specific weekdays instead of on a daily basis [28]. These discrepancies in education provided at ANC are a common phenomenon [29]. Adherence to guidelines, on-the-job training, and specific training on anemia in pregnancy among ANC providers could significantly improve the situation [10,11].

The provision of dietary education is one of the themes for anemia prevention and correction during the ANC [23]. However, this survey revealed that 74% of pregnant women who attended were informed of types of food that prevent anemia in pregnancy. It is advised for pregnant women to consume animal-rich foods, such as meat, liver, and sardines. This is because they are rich in iron, and eating enough green leafy vegetables and fruits rich in vitamin C can enhance the absorption of iron and improve hemoglobin levels. Dietary training is associated with the correction and improvement of hemoglobin levels among pregnant women [30,31]. On the other hand, numerous studies have revealed inadequate basic dietary education, information, or advice to pregnant women [32,33,34]. The provision of accurate dietary information based on the available food supply provides realistic results in anemia prevention [35]. Deworming is another area of education that is important for anemia reduction. However, this study revealed education on deworming was poorly covered among participants. Only 20.8% and 24.8% received information about worm infestation and deworming, respectively. Given such low coverage and the need for deworming, uptake for deworming is very low (5.6%) in Tanzania, as seen in another study [36].

On the other hand, the provision of education on the usage of ITN was high (76%). This aligns with adherence to the ITN preventive strategy observed in the study carried out in Mwanza (88.7%). ITN usage is essential for the prevention of Malaria among pregnant women, who are at increased risk of acquiring the disease. Malaria is mentioned as one of the culprits and a factor in the high prevalence of anemia in Tanzania [18].

Further, our findings reveal women with higher attendance to ANC tend to receive more education on anemia. On the contrary, those with low ANC attendance tend to receive less education on anemia. If women are encouraged to attend more frequent ANC visits, they would not be susceptible to anemia. The provision of education on anemia at ANC significantly reduces the chances of women having anemia. This is supported by a study comparing two Tanzania Demographic and Health Surveys (TDHS), which concluded that anemia is more likely in women with low frequency of ANC attendance [18]. Apart from the education on anemia, ANC offers other services to detect and correct anemia [17,36]. Hence, late booking and poor attendance to ANC put women at risk of anemia [17,30,36].

Further, our study shows that an increase in age increases the chances of receiving health education on anemia at ANC. This could be explained by the fact that women at an older age have more life skills and cumulative knowledge. Younger age and pregnancy at this tender age deny women opportunities to stay in school and accumulate knowledge, skills, and competencies to master life’s skills, including having better nutrition [18,37]. Further, younger age puts women through many other demographic disadvantages, including the inability to demand information and/or services [37,38].

This was a cross-sectional study, and its strength lies in the fact that it was able to measure multiple components of anemia education provisions. However, the cross-section survey checked women’s knowledge of anemia as a proxy for what was taught at ANC services. A gap in interpretation and assimilation of knowledge taught could arise. However, the study was done on women exiting ANC care, hence providing more recently learned information.

## 5. Conclusions

The study reveals limited education provision on anemia among pregnant women. There is a discrepancy in topic coverage among facilities and individuals. Women who frequent the ANC more often have higher chances of receiving a complete package of health education on anemia than those who do not. Further, older women are shown to acquire more education on anemia than their younger counterparts. Significant differences in topic coverage on anemia education exist in this setting.

Restructuring health education sessions and developing teaching aids that will govern coverage of each important topic could help reduce variation in service provision between facilities.

This study used exit interviews of women to examine education provided by health care. However, research utilizing “simulated clients” is recommended.

## Figures and Tables

**Table 1:** Sociodemographic characteristics of respondents.

Characteristic	Group	Number of Participants (%)
Age Group	15–19 years	28 (6.51)
	20–29 years	258 (60.00)
	30–39 years	129 (30.00)
	40–49 years	15 (3.20)
Education Level	Primary level	295 (68.60)
	Secondary level	113 (26.28)
	Tertiary level	22 (5.12)
Economic Activity	Public servant	20 (4.65)
	Subsistence farmer	91 (21.16)
	Housewife	130 (30.23)
	Self-employed (business)	189 (43.95)
ANC Visits	First visit	120 (27.91)
	Second visit	66 (15.35)
	Third visit	105 (24.42)
	Fourth visit	90 (20.93)
	Fifth visit and above	49 (11.40)

**Table 2:** Provision of anemia health education components (topics) during ANC visits.

Characteristics	Overall N (%)
Component of anemia education	
General talk about anemia prevention	302 (70.4)
Minimum required hemoglobin level in pregnancy	201 (46.7)
Signs of moderate/severe anemia in pregnancy	253 (58.8)
Consequences of anemia in pregnancy	234 (54.4)
Types of food to prevent anemia	323 (75.1)
Information about worm infestation	120 (27.9)
Information about deworming	191 (44.4)
Information about malaria prevention (ITN ^1^ and IPT ^2^)	331 (77.0)
Information about iron supplements	216 (50.2)
Provision of anemia different topics	
No anemia topic discussed	27 (6.3)
One topic discussed	22 (5.6)
Two topics discussed	28 (16.0)
Three topics discussed	51 (16.8)
Four topics discussed	50 (20.8)
Five topics discussed	54 (12.0)
Six topics discussed	52 (12.0)
Seven topics discussed	51 (6.4)
Eight topics discussed	54 (4.0)
Nine topics discussed	41 (9.5)
Scores of topics	
0–4 topics	178 (41.4)
5–8 topics (50%)	252 (58.6)

^1^ Insecticide-treated nets; ^2^ intermittent prophylaxis treatment.

**Table 3:** Factors associated with the reported provision of anemia education.

Variable	Bivariate Analysis OR (95% CI ^1^)	*p*-Value	Multivariate Analysis OR (95% CI)	*p*-Value
Age group				
<30 years	Ref			
≥30 years	1.39 [0.92–2.11]	0.115	2.59 [1.07–6.23]	0.034
Education level				
No formal & primary	Ref			
Secondary & tertiary	1.65 [1.07–2.51]	0.022	2.23 [1.37–3.59]	0.001
Occupation				
Housewife	Ref			
Public servant	1.34 [0.52–3.45]	0.544	1.09 [0.39–3.07]	0.869
Farmer	2.59 [1.48–4.58]	0.001	3.26 [1.77–5.98]	<0.001
Vendor/entrepreneur	1.70 [1.09–1.29]	0.021	1.72 [1.07–2.76]	0.024
ANC visits				
First visit	Ref		Ref	
Second visit	1.39 [0.76–2.54]	0.285	1.51 [0.81–2.83]	0.195
Third visit	3.12 [1.80–5.43]	0.000	3.36 [1.88–5.99]	<0.001
Fourth visit	2.61 [1.48–4.61]	0.001	2.38 [1.32–4.26]	0.004
Fifth visit and above	2.46 [1.23–4.91]	0.011	2.75 [1.35–5.62]	0.005

^1^ Confidence interval.

## Data Availability

All data and information are provided within the tables and other details included in the manuscript.

## References

[1] World Health Organization (2008). Worldwide Prevalence of Anaemia 1993–2005.

[2] Bailit J.L., Doty E., Todia W. (2007). Repeated hematocrit measurements in low-risk pregnant women. J. Reprod. Med..

[3] Stoltzfus R.J., Mullany L., Black R.E., Ezzati M., Lopez A.D., Rodgers A., Murray C.J.L. (2004). Comparative Quantification of Health Risks: Global and Regional Burden of Disease Attributable to Selected Major Risk Factors.

[4] Brabin B.J., Hakimi M., Pelletier D. (2001). An analysis of anemia and pregnancy-related maternal mortality. J. Nutr..

[5] Ren A., Wang J., Ye R.W., Li S., Liu J.M., Li Z. (2007). Low first-trimester hemoglobin and low birth weight, preterm birth and small for gestational age newborns. Int. J. Gynecol. Obstet..

[6] Zhao B., Sun M., Wu T., Li J., Shi H., Wei Y. (2024). The association between maternal anemia and neonatal anemia: A systematic review and meta-analysis. BMC Pregnancy Childbirth.

[7] Kharate M.A., Choudhari S.G. (2024). Effects of Maternal Anemia Affecting Fetal Outcomes: A Narrative Review. Cureus.

[8] World Health Organization (2024). Guideline on Haemoglobin Cutoffs to Define Anaemia in Individuals and Populations.

[9] World Health Organization (2020). WHO Guideline on Use of Ferritin Concentrations to Assess Iron Status in Populations.

[10] Stephen G., Mgongo M., Hussein Hashim T., Katanga J., Stray-Pedersen B., Msuya S.E. (2018). Anaemia in pregnancy: Prevalence, risk factors, and adverse perinatal outcomes in Northern Tanzania. Anemia.

[11] Msuya S.E., Hussein T.H., Uriyo J., Sam N.E., Stray-Pedersen B. (2011). Anaemia among pregnant women in northern Tanzania: Prevalence, risk factors and effect on perinatal outcomes. Tanzan. J. Health Res..

[12] Kidanto H.L., Mogren I., Lindmark G., Massawe S., Nystrom L. (2009). Risks for preterm delivery and low birth weight are independently increased by severity of maternal anaemia. S. Afr. Med. J..

[13] Bwana V.M., Rumisha S.F., Mremi I.R., Lyimo E.P., Mboera L.E.G. (2019). Patterns and causes of hospital maternal mortality in Tanzania: A 10-year retrospective analysis. PLoS ONE.

[14] Singal N., Setia G., Taneja B.K., Singal K.K. (2018). Factors associated with maternal anaemia among pregnant women in rural India. Bangladesh J. Med. Sci..

[15] Shija A.E., Rumisha S.F., Oriyo N.M., Kilima S.P., Massaga J.J. (2019). Effect of Moringa Oleifera leaf powder supplementation on reducing anemia in children below two years in Kisarawe District, Tanzania. Food Sci. Nutr..

[16] Ngasala B., Matata F., Mwaiswelo R., Mmbando B.P. (2019). Anemia among schoolchildren with malaria and soil-transmitted helminth coinfections after repeated rounds of mass drug administration in Muheza district, Tanzania. Am. J. Trop. Med. Hyg..

[17] Ministry of Health, Community Development, Gender, Elderly and Children, Ministry of Health, National Bureau of Statistics, Office of Chief Government Statistician, ICF Tanzania Demographic and Health Survey and Malaria Indicator Survey 2015–2016. https://dhsprogram.com/pubs/pdf/fr321/fr321.pdf.

[18] Sunguya B.F., Ge Y., Mlunde L., Mpembeni R., Leyna G., Huang J. (2021). High burden of anemia among pregnant women in Tanzania: A call to address its determinants. Nutr. J..

[19] Solnes Miltenburg A., van der Eem L., Nyanza E.C., van Pelt S., Ndaki P., Basinda N., Sundby J. (2017). Antenatal care and opportunities for quality improvement of service provision in resource limited settings: A mixed methods study. PLoS ONE.

[20] Bintabara D., Nakamura K., Ntwenya J., Seino K., Mpondo B.C.T. (2019). Adherence to standards of first-visit antenatal care among providers: A stratified analysis of Tanzanian facility-based survey for improving quality of antenatal care. PLoS ONE.

[21] Council K.D. Kigoma District Council Statistics. https://www.kigomadc.go.tz/.

[22] Ministry of Finance and Planning, National Bureau of Statistics, Presidents’ Office—Finance and Planning, Office of the Chief Government Statistician (2022). The 2022 Population and Housing Census: Age and Sex Distribution Report.

[23] Ministry of Health, Community Development, Gender, Elderly and Children (2018). Antenatal Care Guidelines.

[24] Reynolds N., Diamantopoulos A., Schlegelmilch B. (1993). Pre-testing in questionnaire design: A review of the literature and suggestions for further research. Mark. Res. Soc. J..

[25] Widyawati W., Jans S., Utomo S., van Dillen J., Janssen A. (2015). A qualitative study on barriers in the prevention of anaemia during pregnancy in public health centres: Perceptions of Indonesian nurse-midwives. BMC Pregnancy Childbirth.

[26] Sunuwar D.R., Sangroula R.K., Shakya N.S., Yadav R., Chaudhary N.K., Pradhan P.M.S. (2019). Effect of nutrition education on hemoglobin level in pregnant women: A quasi-experimental study. PLoS ONE.

[27] Saronga N., Burrows T.L., Collins C.E., Mosha I.H., Sunguya B.F., Rollo M.E. (2020). Nutrition services offered to pregnant women attending antenatal clinics in Dar es Salaam, Tanzania: A qualitative study. Midwifery.

[28] Conrad P., Schmid G., Tientrebeogo J., Moses A., Kirenga S., Neuhann F., Müller O., Sarker M. (2012). Compliance with focused antenatal care services: Do health workers in rural Burkina Faso, Uganda and Tanzania perform all ANC procedures?. Trop. Med. Int. Health.

[29] Von Both C., Fleβa S., Makuwani A., Mpembeni R., Jahn A. (2006). How much time do health services spend on antenatal care? Implications for the introduction of the focused antenatal care model in Tanzania. BMC Pregnancy Childbirth.

[30] Ikeanyi E., Ibrahim A. (2015). Does antenatal care attendance prevent anemia in pregnancy at term?. Niger. J. Clin. Pract..

[31] Shah S., Sharma G., Shris L., Shah S., Sharma M., Sapkota N. (2017). Knowledge on dietary patterns among pregnant women attending antenatal care check-up in Narayani hospital, Nepal. Int. J. Community Med. Public Health.

[32] Lucas C., Charlton K.E., Yeatman H. (2014). Nutrition advice during pregnancy: Do women receive it and can health professionals provide it?. Matern. Child Health J..

[33] Morales S., Kendall P.A., Medeiros L.C., Hillers V., Schroeder M. (2004). Health care providers’ attitudes toward current food safety recommendations for pregnant women. Appl. Nurs. Res..

[34] de Jersey S.J., Nicholson J.M., Callaway L.K., Daniels L.A. (2013). An observational study of nutrition and physical activity behaviours, knowledge, and advice in pregnancy. BMC Pregnancy Childbirth.

[35] Munyogwa M.J., Gibore N.S., Ngowi A.F., Mwampagatwa I.H. (2021). Effect of nutritional education intervention to reduce anaemia during pregnancy in Dodoma City, Tanzania: Protocol for a cluster randomized controlled trial. Biol. Methods Protoc..

[36] Konje E.T., Ngaila B.V., Kihunrwa A., Mugassa S., Basinda N., Dewey D. (2022). High Prevalence of Anemia and Poor Compliance with Preventive Strategies among Pregnant Women in Mwanza City, Northwest Tanzania: A Hospital-Based Cross-Sectional Study. Nutrients.

[37] Rahman M.A., Rahman M.S., Aziz Rahman M., Szymlek-Gay E.A., Uddin R., Islam S.M.S. (2021). Prevalence of and factors associated with anaemia in women of reproductive age in Bangladesh, Maldives and Nepal: Evidence from nationally-representative survey data. PLoS ONE.

[38] Massawe S., Ronquist G., Nyström L., Lindmark G. (2002). Iron status and iron deficiency anaemia in adolescents in a Tanzanian suburban area. Gynecol. Obstet. Investig..

